# Human Rotavirus Serotype G9, São Paulo, Brazil, 1996–2003

**DOI:** 10.3201/eid1206.060307

**Published:** 2006-06

**Authors:** Rita Cássia Compagnoli Carmona, Maria do Carmo Sampaio Tavares Timenetsky, Simone Guadagnucci Morillo, Leonardo José Richtzenhain

**Affiliations:** * Adolfo Lutz Institute, São Paulo, Brazil;; † University of São Paulo, São Paulo, Brazil

**Keywords:** rotavirus, diarrhea, molecular epidemiology, genotypes, research

## Abstract

A total of 3,101 fecal specimens were collected during an 8-year survey for rotavirus infection in São Paulo, Brazil. Group A rotavirus was detected in 774 (25.0%) specimens. Of these, 431 strains (55.7%) were analyzed for G and P types by reverse transcription–polymerase chain reaction; G1 was the predominant serotype (68.2%), followed by G9 (17.2%), G4 (6.3%), G2 (1.2%), G3 (0.7%), mixed infection (1.8%), and untypeable (4.6%). Both rotavirus G and P types could be established in 332 strains (77.0%). We identified the 4 most common strains worldwide: P[8]G1 (66.6%), P[4]G2 (1.0%), P[8]G3 (0.6%), and P[8]G4 (7.2%). Among the single G9 strains detected, VP4 genotyping showed that P[8]G9 was the most prevalent, followed by P[4]G9 and P[6]G9. The emergence and high frequency of rotavirus G9 in São Paulo, Brazil, and other parts of the world will affect the development and evaluation of future vaccines.

## Abstract 2

Diverse rotavirus strains are present, and frequency of G9 is high.

Group A rotavirus is the most common cause of acute gastroenteritis in infants and young children worldwide (*1*). More than 130 million cases of diarrhea each year are attributed to rotavirus. It is estimated to cause >400,000 deaths annually in children <5 years of age and is responsible for 2 million hospital admissions due to acute diarrhea worldwide. In developing countries, an estimated 1,205 children die from rotavirus disease each day, and 82% of these deaths occur in children in the poorest countries (*2*)

Rotavirus serotypes are determined by neutralizing antibody responses to each of the 2 outer capsids proteins, VP7 (G serotype) and VP4 (P serotype) (*1*). To date, 11 VP7 G serotypes and 13 P serotypes have been identified in humans. Serotypes G1, G2, G3, and G4 are frequently associated with diarrhea in humans and have become prime targets for vaccine development (*3**,**4*). The recent emergence and wide distribution of rotavirus G9 indicate that this serotype may become the fifth relevant strain (*5**,**6*). Unusual types of rotavirus have been described in certain settings. The G5 type has been reported in Brazil, Argentina, Paraguay, Cameroon, and the United Kingdom (*6**,**7*); G6 has been detected in Italy, Australia, India, the United States, Belgium, and Hungary; G8 has been frequently isolated in Africa and sporadically in other countries; rotavirus G10 specificity has been reported in the United Kingdom, India, Thailand, Paraguay, and Brazil (*6*); G11 type was recently detected in Dhaka, Bangladesh (*4*); and the G12 type has been detected in the Philippines (*8*), Thailand (*9*), the United States (*10*), India (*11*), Japan (*12*), Korea (*13*), Argentina (*14*), and Brazil (*15*).

The genotypes VP4 P[8] and P[4] are the most common P types that infect humans. The P[8] type is generally associated with VP7 types G1, G3, and G4, and the P[4] type is associated with G2 (*16*). Combined G and P genotyping may have advantages in identifying reassortants as unusual or new virus strains (*17*). Continued surveillance of the diverse rotavirus strains circulating in a community is crucial before developing a vaccine and during and after implementing an immunization program. Therefore, we describe the results of an 8-year surveillance study of G- and P-type rotavirus strains from persons with acute diarrhea in the state of São Paulo, Brazil.

## Materials and Methods

**Figure 1:**
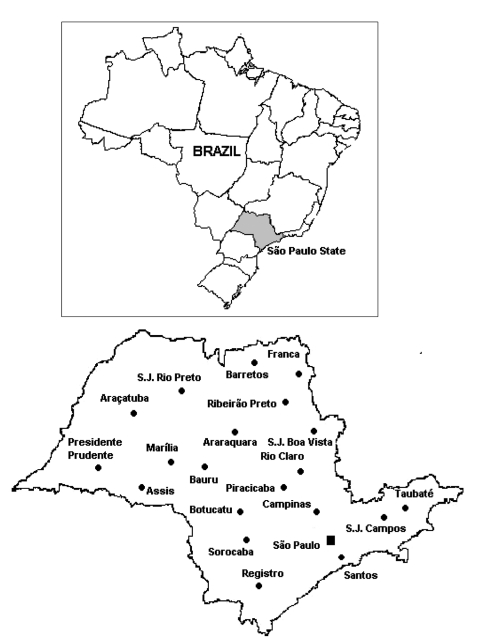
Map of São Paulo State, Brazil, indicating where fecal specimens were collected during the 8-year survey period.

From 1996 to 2003, a total of 3,101 fecal specimens were collected from children <5 years of age, school-age children (5–17 years), adults (18–59 years), and elderly patients (>60 years) with acute gastroenteritis. These patients received treatment for diarrhea at the departments of public health or were admitted to hospitals in several cities in São Paulo State, in southeast Brazil. São Paulo State has an area of ≈248,800 km^2^ and a population of 40 million (21.5% of the population of Brazil). Figure 1 shows main cities in São Paulo where samples were collected. Epidemiologic data (age, date of diarrhea onset, date of sample collection) were available from some patients. Specimens were stored at –20°C until tested for rotavirus and characterized. Study methods were approved by the ethical committee of Adolfo Lutz Institute.

All specimens were screened for rotavirus by using a commercial enzyme-linked immunosorbent assay (ELISA) (Premier Rotaclone, Meridian Diagnostics, Cincinnati, OH, USA) with monoclonal antibodies specific for group A human rotavirus, according to the manufacturer's protocol. Rotavirus double-stranded RNA (dsRNA) was extracted directly from stool by the TRIzol method (Invitrogen, Carlsbad, CA, USA) and precipitated with isopropanol. The extracted dsRNA was subjected to G and P typing by multiplex reverse transcription–polymerase chain reaction (RT-PCR) with type-specific primers. Consensus primers Beg9 and End9 were used in a first-round PCR (30 cycles) to amplify the full-length VP7 gene (1,062 bp); cDNA was used in a second-round PCR for G typing (25 cycles) with primer set aBT1 (G1), aCT2 (G2), aET3 (G3), aDT4 (G4), aFT9 (G9) and primer set FT5 (G5), DT6 (G6), HT8 (G8), ET10 (G10), BT11 (G11) (*18**,**19*). For P typing, consensus primers Con2 and Con3 were used in a first-round RT-PCR (30 cycles) to amplify the 876 bp of the VP8* region of the VP4 gene, and the second-round PCR (20 cycles) used primer set 1T-1 (P[8]), 2T-1 (P[4]), 3T-1 (P[6]), 4T-1 (P[9]), 5T-1 (P[10]) (*20*). All PCR products were analyzed by electrophoresis in 1.2% agarose gels, containing 0.5 µg ethidium bromide per milliliter and visualized under UV illumination.

## Results

**Figure 2:**
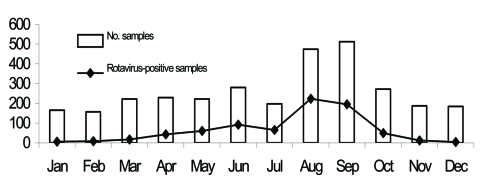
Temporal distribution of rotavirus strains from children, adults, and elderly patients with acute diarrhea, São Paulo, Brazil, 1996–2003.

**Figure 3:**
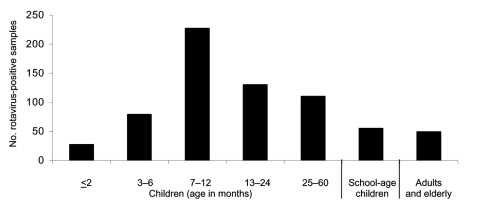
Rotavirus infection among children (<5 years of age), school-age children (5–17 years), adults (18–59 years), and elderly patients (>60 years) with acute diarrhea, São Paulo, Brazil, 1996–2003.

Rotavirus was detected in 774 (25.0%) of 3,101 specimens collected from children, adults, and elderly patients in São Paulo during an 8-year period. Rotavirus infection was found predominantly in the winter and in drier months. The incidence peaked in August (Figure 2). The age or date of birth was provided for 677 (87.5%) of 774 patients who tested positive for rotavirus. Rotavirus disease was detected mainly in children <2 years of age (463 [59.8%] of 774), and peaks of incidence occurred from 7 to 12 months (Figure 3). However, rotavirus infection also was detected in adults and elderly patients (55 [7.1%] of 774).

We randomly selected 431 rotavirus-positive samples (55.7%) for determination of G and P genotypes by an RT-PCR assay. G1 was the predominant serotype in these samples (294, 68.2%), followed by G9 (74, 17.2%), G4 (27, 6.3%), G2 (5, 1.2%), G3 (3, 0.7%), mixed infection (8, 1.8%), and untypeable (20, 4.6%) (Table 1). The distribution of rotavirus types in São Paulo during this 8-year period shows that G1 was the most prevalent genotype in most years, but it was displaced by G9 in 2002. Incidence of G2 and G3 serotypes was low during the period of analysis. Frequency of G4 serotype differed during the surveillance period; it was not detected in 1996 and 2003. We found several mixed infections from 2000 to 2003 (G1+G4, G1+G9, G2+G3, and G4+G9).

**Table 1:** Distribution of rotavirus G types from children, adults, and elderly patients with acute diarrhea in São Paulo, Brazil, 1996–2003

Year	No. rotavirus isolates	No. (%) selected for genotyping	G1	G2	G3	G4	G9	Mixed*	Not typeable
1996	33	21 (63.3)	19 (90.5)	2 (9.5)	0	0	0	0	0
1997	121	48 (39.7)	45 (93.8)	0	0	3 (6.3)	0	0	1 (2.1)
1998	45	16 (35.6)	12 (75.0)	0	0	4 (25.0)	0	0	0
1999	99	56 (56.6)	46 (82.1)	0	0	10 (17.9)	0	0	2 (3.6)
2000	98	52 (53.1)	42 (80.8)	0	0	0	7 (13.4)	3 (5.8)	5 (9.6)
2001	57	46 (80.7)	28 (60.9)	2 (4.3)	1 (2.2)	6 (13.0)	4 (8.7)	1 (2.2)	1 (2.2)
2002	90	49 (54.4)	18 (36.7)	0	2 (4.1)	4 (8.2)	23 (46.9)	2 (4.1)	6 (12.2)
2003	231	127 (55.0)	84 (66.1)	1 (0.8)	0	0	40 (31.5)	2 (1.6)	5 (3.9)
Total	774	431 (55.7)	294 (68.2)	5 (1.2)	3 (0.7)	27 (6.3)	74 (17.2)	8 (1.8)	20 (4.6)

*Mixed infections: 2000, G1+G9 (n = 3); 2001, G2+G3 (n = 1); 2002, G1+G9 (n = 1) and G4+G9 (n = 1); 2003, G1+G9 (n = 2).

Both rotavirus G and P types could be established in 332 (77.0%) strains, and 17 different P and G associations were detected (Table 2). Of these, we identified the 4 most globally common strains, P[8]G1, P[8]G4, P[4]G2, and P[8]G3, which represented 75.3% of all typed rotavirus strains. Uncommon strains were also detected, including P[8]G9, P[4]G9, P[6]G9, P[4]G1, P[6]G1, P[6]G2, and P[4]G4. And combination P-G mixed infections were as diverse as P[8]G1+G4, P[8]G1+G9, P[6]G1+G9, P[4]G2+G3, and P[8]G4+G9.

**Table 2:** Association of P- and G-type rotavirus strains from patients with acute diarrhea, São Paulo, Brazil, 1996–2003

P and G association	1996, n (%)	1997, n (%)	1998, n (%)	1999, n (%)	2000, n (%)	2001, n (%)	2002, n (%)	2003, n (%)	Total, n (%)
Common genotypes	17 (94.4)	32 (100)	15 (93.8)	50 (98.0)	34 (81.0)	21 (72.4)	16 (38.1)	65 (63.7)	250 (75.3)
P[8]G1	15 (83.3)	30 (93.7)	12 (75.0)	40 (78.4)	34 (81.0)	14 (48.3)	12 (28.5)	64 (62.7)	221 (66.6)
P[4]G2	2 (11.1)	0	0	0	0	0	0	1 (1.0)	3 (1.0)
P[8]G3	0	0	0	0	0	0	2 (4.8)	0	2 (0.6)
P[8]G4	0	2 (6.3)	3 (18.8)	10 (19.6)	0	7 (24.1)	2 (4.8)	0	24 (7.2)
Uncommon genotypes	1 (5.6)	0	1 (6.2)	1 (2.0)	0	6 (20.7)	3 (7.1)	1 (1.0)	13 (3.9)
P[4]G1	1 (5.6)	0	0	0	0	2 (7.0)	2 (4.8)	1 (1.0)	6 (1.8)
P[6]G1	0	0	0	0	0	1 (3.4)	0	0	1 (0.3)
P[4]+P[6]G1	0	0	0	0	0	0	1 (2.4)	0	1 (0.3)
P[6]+P[8]G1	0	0	0	1 (2.0)	0	1 (3.4)	0	0	2 (0.6)
P[6]G2	0	0	0	0	0	2 (7.0)	0	0	2 (0.6)
P[4]G4	0	0	1 (6.2)	0	0	0	0	0	1 (0.3)
G9 genotypes	0	0	0	0	5 (12.0)	1 (3.4)	21 (50.0)	34 (33.3)	61 (18.4)
P[4]G9	0	0	0	0	0	0	1 (2.4)	3 (2.9)	4 (1.2)
P[6]G9	0	0	0	0	0	0	0	1 (1.0)	1 (0.3)
P[8]G9	0	0	0	0	5 (12.0)	1 (3.4)	20 (47.6)	30 (29.4)	56 (16.9)
Mixed infection					3 (7.0)	1 (3.4)	2 (4.8)	2 (2.0)	8 (2.4)
P[8]G1+G9	0	0	0	0	3 (7.0)	0	0	1 (1.0)	5 (1.5)
P[6]G1+G9	0	0	0	0	0	0	1 (2.4)	0	1 (0.3)
P[4]G2+G3	0	0	0	0	0	1 (3.4)	0	0	1 (0.3)
P[8]G4+G9	0	0	0	0	0	0	1 (2.4)	0	1 (0.3)

## Discussion

We detected rotaviruses in the specimens of 25.0% of patients with acute diarrhea, which is comparable to the prevalence seen in other studies in Brazil (*21*). Among children <5 years of age, we detected rotavirus infection mainly in those <2 years of age (81.0%) (data not shown); in adults, rotavirus was detected less frequently (7.1%). The finding of a low percentage of rotavirus infection among adults is likely because the disease is generally perceived to be a childhood infection (*22*). Common epidemiologic settings for rotavirus infection among adults include endemic disease, epidemic outbreak, travel-related infection, and child-to-adult transmission (*23*).

In studies performed at various locations in Brazil with diverse climatic conditions, rotavirus disease appears to occur year-round (*24*). In São Paulo State, however, infection occurred mainly during cooler and drier seasons; similar observations have been made in other countries with temperate climates (*16*).

During the 8-year period studied, the G1 type was the most prevalent rotavirus strain. The second most prevalent was the G9 type, which accounted for 17.2% of disease, followed by G4, G2, and G3, which are common around the world. G1 was the most prevalent type in most years; however, it was displaced by G9 during the 2002 season, when G9 accounted for 46.9% of typed isolates. The G9 type has been reported to be a common cause of diarrhea and has become the fifth most common serotype, which suggests that it may be a substantial cause of diarrhea in humans (*5**,**6*). This type has been detected in Brazil since 1997 (*25**,**26*).

Surveillance on rotavirus types has been performed in São Paulo for >18 years, from 1986 to 2003 (*27**,**28*). The first G9 type was isolated in 2000 and has been fluctuating in frequency since its emergence (Table 1). The G5 type, normally associated with animal rotavirus (pigs and horses), has been frequently detected in persons in Brazil and it was considered an endemic virus (*28**,**29*). Nevertheless, in our survey in São Paulo, G5 rotavirus was not detected. Its incidence in Brazil has been decreasing over the last few years, and it may be disappearing (*25**,**26*); this type of rotavirus is likely to be a cyclic form. G9 strains have also been detected in animals (lambs and pigs [1]); detection of animal rotavirus provides evidence for natural human-animal genetic reassortment (*30*). Surveillance programs for animal rotavirus may aid in the development of next-generation vaccines (*6*).

Characterization of rotavirus VP4 types showed various strains. In this study, the 4 most globally common strains, P[8]G1, P[4]G2, P[8]G3, and P[8]G4, represented 75.3% of all typed viruses. The most prevalent association was P[8]G1, followed by P[8]G9. Worldwide, the 4 predominant rotavirus genotypes make up nearly 90% of all rotavirus infections (*16*). In Brazil, epidemiologic data on the prevalence of G and P types have been collected since the 1980s (*27*). This study showed great diversity of rotavirus strains in São Paulo. The uncommon genotypes P[4]G1, P[4]G4, P[4]+P[6]G1, and P[6]+P[8]G1 were also seen in some cases, similar to results from other countries (*31**,**32*). In our study, the uncommon genotypes P[6]G1, P[6]G2, and P[6]G9 were detected in children with acute diarrhea with an average age of 14 months (data not shown). Many studies have shown that this type is often detected in very young children with diarrhea, which suggests that P[6] strains may promote infection at an early age. Originally, P[6] in association with rotavirus types G1–G4 was detected in asymptomatic neonates (*33*), but recent studies have also shown that P[6]G9 circulates in hospitalized children without diarrhea (*34*). These strains are considered naturally attenuated and have been used to develop a vaccine candidate. Worldwide, rotavirus strains with the P[6] genotype have been seen in children with diarrhea (*35**–**37*). In Brazil, other studies isolated the P[6] type from children with acute diarrhea (*26**–**28*). In our survey, several mixed G and P types also appeared but in a low percentage (2.6%). The detection of unusual strains and mixed infections in this study suggests a previously unrecognized diversity among Brazilian rotavirus infections (*28*).

Among the single G9 strains detected in this survey, VP4 genotyping showed that P[8]G9 was the most prevalent (75.7%), followed by P[4]G9 (5.4%) and P[6]G9 (1.4%) (data not shown). This diversity among G9 types has also been detected in other studies (*5**,**6**,**26*). Combined data from P and G typing are relevant to identify new strains that might have resulted from reassortment of genes between diverse human-human and human-animal rotaviruses (*38*).

Recently, 2 live rotavirus oral vaccines have been licensed in some countries and made available on the market, including a monovalent vaccine derived from the most common human rotavirus strain, P[8]G1, and a pentavalent vaccine based on a bovine strain, WC3, that contains 5 human-bovine reassortant viruses (G1, G2, G3, G4, and P[8]). Both vaccines have shown efficacy against severe rotavirus disease (*39**,**40*). In August 2005, the live, attenuated P[8]G1 human rotavirus vaccine was licensed in Brazil, the first country to introduce this vaccine into the public health network.

Our data show that challenges exist for the design of rotavirus vaccine for the Brazilian population and underscore that virus strain surveillance should be ongoing. Surveillance programs can establish whether G9 rotavirus strains will continue to rise in prevalence or whether they will follow a cyclical pattern of emergence, as has been shown for G1–G4. The composition of future rotavirus vaccines is likely to be formulated according to the geographic setting and the distribution of G and P strains.
